# How to irrigate the eye

**Published:** 2017-02-10

**Authors:** Sue Stevens

**Affiliations:** 1 Former Nurse Advisor, Community Eye Health Journal, International Centre for Eye Health, London School of Hygiene and Tropical Medicine, London, UK.

Remember to wash your hands before and after performing all procedures.

## Indications

- To remove single or multiple foreign bodies from the eye
- To wash the eye thoroughly following any chemical injury to the eye

**Note:** Irrigation of the conjunctival sac is an emergency treatment if there has been chemical injury to the eye.

Alkali (e.g. lime) and acid (e.g. car battery) solutions in the eye may cause serious damage to the cornea and conjunctiva, resulting in long-term loss of vision.

The sooner the chemical can be diluted and removed, the less likely there is to be damage to the ocular surface.

**Immediate, copious irrigation may save the eye after chemical injury.**

- For foreign body removal, a minute or so of irrigation should be sufficient to remove any foreign bodies.
- For severe acid or alkali burns, emergency irrigation should continue for **at least** 15 minutes; 30 minutes is better. It is advisable to continue to irrigate acid/alkali burn injuries for a further 12–24 hours by setting up a saline drip to continue to gently irrigate the eye.

## You will need:

- A large syringe or a small receptacle with a pouring spout, such as a feeding cup
- Irrigating fluid (normal saline or clean water at room temperature)
- Local anaesthetic eye drops
- Towel or gauze swabs
- Lid retractors if available
- A bowl or kidney dish

## Method

- Instil local anaesthetic eye drops.
- With the patient lying down, protect the neck and shoulders with a towel or sheet.
- Place the bowl or kidney dish against the cheek, on the affected side, with the head tilted sideways towards it.
- Fill the feeding cup or syringe with the irrigating fluid and test the temperature on your hand.
- Ask the patient to fix his/her gaze ahead.
- Open the eyelids. If necessary, **gently** use eyelid retractors.
- Pour or syringe the fluid slowly and steadily, from no more than 5 centimetres away, onto the front surface of the eye, inside the lower eyelid and under the upper eyelid.
- If possible, evert the upper eyelid to access all of the upper conjunctival fornix.
- Ask the patient to move the eye in all directions while the irrigation is maintained.
- Check and record the visual acuity when the procedure is finished.
- In alkali and acid burns, refer the patient to an ophthalmologist for assessment.

**Figure:**
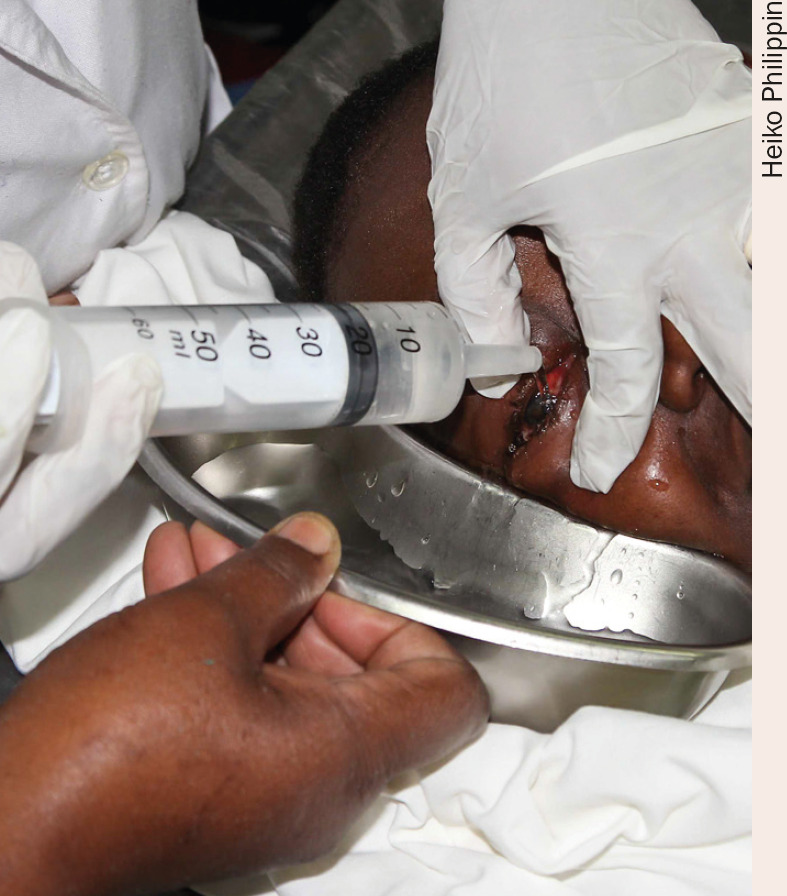
Irrigating the eye

